# Impact of orange essential oil on enteric methane emissions of heifers fed bermudagrass hay

**DOI:** 10.3389/fvets.2022.863910

**Published:** 2022-08-16

**Authors:** Rafael Jiménez-Ocampo, María D. Montoya-Flores, Gerardo Pamanes-Carrasco, Esperanza Herrera-Torres, Jacobo Arango, Mirna Estarrón-Espinosa, Carlos F. Aguilar-Pérez, Elia E. Araiza-Rosales, Maribel Guerrero-Cervantes, Juan C. Ku-Vera

**Affiliations:** ^1^Laboratory of Climate Change and Livestock Production, Department of Animal Nutrition, Faculty of Veterinary Medicine and Animal Science, University of Yucatan, Mérida, Yucatan, Mexico; ^2^National Institute of Research in Forestry, Agriculture and Livestock-INIFAP, Experimental Field Valle del Guadiana, Durango, Mexico; ^3^National Center for Disciplinary Research in Physiology and Animal Breeding, National Institute for Forestry, Agriculture and Livestock Research-INIFAP, Queretaro, Mexico; ^4^Institute of Silviculture and Wood Industry, National Council of Science and Technology—Durango State Juarez University, Durango, Mexico; ^5^National Technology of Mexico, Technological Institute of Valle del Guadiana, Durango, Mexico; ^6^Tropical Forage Program—International Center for Tropical Agriculture (CIAT), Palmira, Colombia; ^7^Food Technology Unit, Centro de Investigación y Asistencia en Tecnología y Diseño del Estado de Jalisco, Jalisco, Mexico; ^8^Department of Animal Nutrition, National Council of Science and Technology—Durango State Juarez University, Durango, Mexico; ^9^Department of Small Ruminant Nutrition, Faculty of Veterinary Medicine and Animal Science, Durango State Juarez University, Durango, Mexico

**Keywords:** cattle, enteric methane, essential oil, feed additive, plant secondary metabolites

## Abstract

In this study, the effects of orange essential oil (OEO) on the rumen fermentation, nutrient utilization, and methane (CH_4_) emissions of beef heifers fed a diet of bermudagrass (*Cynodon dactylon*) were examined. In addition, *in vitro* and *in situ* experiments were conducted. The *in vitro* experiment consisted of three treatments: control (CTL, no OEO), OEO1 (0.25% OEO), and OEO2 (0.5% OEO). The forage to concentrate ratio was 70:30 (dry matter [DM] basis) in all treatments. No changes in pH, proportions of volatile fatty acids, and the acetate:propionate ratio were observed (*P* > 0.05). The addition of 0.25% OEO resulted in a reduction in CH_4_ production (mL/g) relative to the control (*P* < 0.05). In the *in situ* experiment, 5 g of total mixed ration (CTL, OEO1, and OEO2) were incubated for 6, 12, 24, 48, and 72 h. Potential and effective degradability were not affected by OEO supplementation (*P* > 0.05). In the *in vivo* study, six crossbred beef heifers (*Bos indicus* × *Bos taurus*), fitted with rumen cannulas, were assigned to three different treatments: no additive (CTL), 0.25% OEO (OEO1), and 0.5% OEO (OEO2) in a replicated 3 × 3 Latin square (21-day periods). Heifers were fed at 2.8% body weight. *In vivo* CH_4_ production was measured in open-circuit respiration chambers. Reductions in gross energy consumption, apparent total tract digestibility, and rumen valerate concentration were observed for OEO2 compared to the control (*P* < 0.05). Additionally, decreases in CH_4_ emissions (g/day; *P* < 0.05) and CH_4_ (MJ gross energy intake/day; *P* < 0.05) were observed in response to supplementation of 0.5% OEO as compared to the CTL treatment. Thus, supplementation of 0.5% OEO reduced CH_4_ emissions (g/day) by 12% without impacting the DM intake of heifers fed bermudagrass hay as a basal ration.

## Introduction

Greenhouse gas (GHG) emissions from livestock production account for 10–12% of global GHG emissions and are expected to increase by 40% by 2050 (1). Methane (CH_4_) emitted by ruminants is a substantial GHG source. Given that CH_4_ is a short-lived GHG, reducing its emissions is important for mitigating the adverse effects of climate change (2, 3). The development of effective CH_4_ mitigation strategies that do not adversely affect animal performance is important, especially in tropical countries where livestock plays an important role in ensuring food security and contributes to poverty reduction. Ideally, GHG mitigation strategies should reduce the environmental impact of livestock farming, enhance the productivity and profitability of production systems, and meet the needs of consumers (4–6). Over 200,000 secondary plant metabolites have been identified as potential modulators of the rumen microbiome, and some of these metabolites can potentially enhance animal production efficiency and reduce energy losses in the form of enteric CH_4_ (7). Essential oils (EOs) are complex mixtures of volatile or aromatic chemicals, and their main constituents are terpenoids (6). Essential oils are present in various parts of plants—such as the roots, peels, seeds, buds, fruit, leaves, twigs, wood, and bark—and are frequently extracted through steam distillation (6–9). Essential oils are generally recognized as safe for mammals and exhibit antimicrobial, antiparasitic, and antifungal activity (5, 10, 11). Furthermore, some EOs possess antioxidant properties and can induce changes in the rumen microbiome, resulting in a reduction in CH_4_, and increases in propionate or rumen bypass protein (12). Although numerous EOs have been studied *in vitro*, only a few EOs have been studied *in vivo* (13). *Citrus sinensis* L. is one of the world's most important fruit crops. In addition to its use as a food source for humans, its by-products, such as peel, seeds, and pulp, account for 50% of the output of orange fruit production (14). Sweet orange essential oil (OEO) is also an important orange by-product and contains numerous different compounds (between 20 and 60) that are mainly present in the flavedo or exocarp.

A large number of compounds contained in OEO, such as monoterpenes (limonene as the main component), sesquiterpenes, and oxygenated derivatives are volatile but also a small portion of non-volatile compounds are contained in OEO (11, 15). Although OEO can reduce CH_4_ production *in vitro* (16), determining whether it has the same effect when used as a feed additive *in vivo* is critically important (16, 17). Wu (17) found that the use of citrus essential oil (D-limonene, 80.13%) in sheep has no adverse effects on body weight (BW), dry matter intake (DMI), average daily gain, and total-tract nutrient digestibility, and that it has intermittent anti-methanogenic effects. Kotsampasi (11) reported that milk yield, milk fat yield, and feed efficiency of ewes were all enhanced when 450 mg/kg OEO (d-limonene, 95.17 g/100 g) was added to a ration with a 57:43 concentrate-to-forage ratio. The replacement of 20% extruded corn with citrus pulp containing bioactive compounds, such as limonene, reduced CH_4_ production in dairy sheep (18). In tropical regions, forages are typically low in crude protein but high in neutral detergent fiber (NDF). Thus, the targeted supplementation of concentrates or the use of feed additives in roughage-based diets could enhance animal performance and promote rural development in the tropics (19). The aim of this study was to evaluate the effect of dietary OEO supplementation on rumen fermentation, nutrient utilization, and enteric CH_4_ production in beef heifers, fed tropical bermudagrass hay as basal ration. In addition, we examined the effect of OEO on total gas and CH_4_ production, and fermentation patterns *in vitro*, as well as potential and effective degradability *in situ*.

## Materials and methods

The experimental procedures were approved by the Bioethics Committee at the University of Yucatan (approval no. CB-CCBA-D-2019-01). The research was carried out at the University of Yucatan's Faculty of Veterinary Medicine and Animal Science's Laboratory of Climate Change and Livestock Production, as well as at the Durango State Juarez University's Faculty of Veterinary Medicine and Animal Science. The experimental ration administered in the *in vivo* experiment was also used as a substrate in the *in vitro* and *in situ* experiments.

### Volatile characterization of OEO

The same batch of OEO was used throughout the study. The OEO was provided by CITROFRUT, S.A. de C.V., Monterrey, NL, Mexico. The composition of the OEO was analyzed using a GC/MSD system (GC 7890B and MSD 5977A, Agilent Technologies, Santa Clara, CA, USA). Separation was achieved using an HP-INNOWAX capillary column (60 m × 0.25 mm ID × 0.25-μm film thickness). The temperatures of the injector and detector were 230 and 260°C, respectively. The initial oven temperature was 60°C. The temperature was increased to 250°C at a rate of 4°C/min and held for 25 min. The carrier gas was helium at a flow rate of 0.8 mL/min. The injection volume was 0.3 μL using a split ratio of 1:200, with a 70 eV ionization potential. The mass spectrometer was operated in SCAN mode, with a scanning range of *m*/*z* 30–480. Volatile components of the OEO were preliminarily identified by matching their mass spectra with those recorded in the NIST14L MS Data Library, as well as by elution order based on published retention index datasets. Identities were also confirmed by the injection of pure compounds obtained from Sigma-Aldrich, USA (95% minimum purity) when possible under the same analytical conditions. Volatile compounds were quantified using the same chromatographic system with flame ionization detection. Chromatographic conditions were the same as those in the GC–MS analysis, with air and hydrogen flows of 400 and 40 mL/min, respectively. Substances were quantified by averaging the area percentage of each component after a duplicate analysis of OEO.

### *In vitro* experiment

#### *In vitro* fermentation patterns

Six heifers (*Bos indicus* × *Bos taurus*) fitted with a permanent rumen cannula (10 cm i.d., Bar Diamond Inc., Parma, ID, USA) were adapted to the three different diets (Table 1) for 15 days, before rumen liquor was collected. The experiment was arranged as a completely randomized design with three treatments: (1) Control (CTL, no additive); (2) OEO1 at 0.25% incubated DM (IDM); and (3) OEO2 at 0.50% IDM. The substrate of each experimental treatment (1 g DM) was incubated at 39°C in ANKOM glass incubators (ANKOM Technology, USA) with 120 mL of ruminal liquor pooled from each pair of heifers and buffer solution in a 2:1 ratio. The modules were opened after 24 h of incubation, and the pH of the incubation fluid was measured immediately and evaluated using a portable pH meter (Hannah^®^ Instruments, Woonsocket, RI, USA). Aliquots of the incubation fluid (10 mL) were collected and mixed with 2.5 mL of metaphosphoric acid [25% weight per volume (w/v)] following a previously described method (20). The volatile fatty acid (VFA) content of the samples was quantified using a gas chromatography system (7820A GC system Agilent Technologies Inc, Santa Clara, CA, USA) equipped with a flame ionization detector. The samples were analyzed in triplicates.

**Table 1:** Ingredient and nutrient composition of the total mixed ration.

**Item**	**Treatments**		
	**CTL**	**OEO1**	**OEO2**
Ingredients (g/kg DM)
Bermudagrass hay, ground	700	700	700
Soybean meal	130	130	130
Ground corn	110	110	110
Sugarcane molasses	40	37	35
Minerals and vitamins premix	20	20	20
Orange essential oil	0.0	2.5	5.0
Chemical composition (g/kg DM, n=3)
Organic matter	916	917	918
Crude protein	105	105	105
Neutral detergent fiber	590	618	607
Acid detergent fiber	364	383	379
Gross energy (MJ/kg DM)	15.1	15.1	15.0

DM, dry matter. The premix contained 24% Ca; 1% P; 1,750 g/kg Zn; 8.83 g/kg Se; 590 mg/kg Cu; 2,120 IU vitamin E and 130 IU vitamin A.

#### *In vitro* gas and CH_4_ production

The *in vitro* incubations were carried out using the ANKOM RF Gas Production System (ANKOM Technology, Macedon, NY, USA). Samples (ca. 1 g DM) from each treatment (CTL, OEO1, and OEO2) were incubated in triplicates in ANKOM glass modules equipped with a pressure transducer. This batch culture procedure was repeated in two consecutive runs on two different days (*n* = 6 per treatment). Fermentations were carried out according to the manufacturer's instructions by incubating the sample with a 2:1 mixture of buffer and ruminal inoculum solution. Four cows (*Bos indicus* × *Bos taurus*) fitted with a permanent rumen cannula (10 cm i.d., Bar Diamond Inc., Parma, ID, USA) were adapted to the diet for 15 days (70:30 forage:concentrate ratio), and rumen liquor was withdrawn from these cows in the morning before they were fed (between 0800 and 0830 h). The ruminal inoculum was collected as a pooled sample and immediately transported in thermos bottles to the laboratory, where it was mixed, flushed with CO_2_, and filtered with four layers of cheesecloth (21). Buffer solutions were prepared following the instructions provided by Ankom (ANKOM Technology, Macedon, NY, USA). The pressure was measured every hour during the 96 h incubation period. The kinetics of *in vitro* gas production were determined by fitting data to the Gompertz function (22) using the following equation:


GP =Gmax∗exp[−A∗ exp(−k∗t)]

where GP = gas production at time *t* (mL); *G*_max_= maximum gas production (mL); *k* = constant gas production rate (h^−1^); and *A* = latency time before gas production begins (h). To measure the proportions of CH_4_ and CO_2_, the pressure relief valve of the modules was opened for 2 s as per the manufacturer's instructions. The expelled gas was sent through a tube to a portable gas analyzer (GEMTM5000, LANDTEC, USA) as per a previously described method (23).

### *In situ* experiment

Six rumen-cannulated heifers were used to estimate *in situ* rumen DM disappearance. Five grams DM of TMR with or without OEO (Table 1) were ground and screened using a 1-mm sieve before being placed in nylon bags (10 × 20 cm, 50 × 10-micron average pore size; R1020 Ankom Technology, Macedon, NY, USA) at each time point. Duplicate samples were incubated in the ventral sac of the rumen. Each heifer (*n* = 6) was considered a replicate. The bags were incubated in the rumen for 0, 12, 24, 48, 72, and 96 h in reverse order to ensure that all bags were removed at the same time. After incubation, the nylon bags were manually cleaned in tap water, dried at 60°C for 48 h, and then weighed for DM determination. The exponential model proposed by Ørskov and McDonald (24) was used to estimate *in situ* degradation curves (DM) as follows:


$$P\;=\text{ a+b }\times \;\left(1^{-exp-ct}\right)$$

where *a* is the soluble and rapidly degradable fraction; *b* is an insoluble but potentially degradable fraction; *c* is the fractional disappearance rate constant at which *b* is degraded, and P is the proportion (%) of dry matter degraded at time *t* (incubation h). The sum of *a* + *b* was used to obtain the potential degradability (PD, %). The following equation was used to estimate effective degradability (ED):


ED = a+ ((b×c)/(c+k))

where *a, b*, and *c* are the same values indicated in the aforementioned equation, and *k* is the predicted passage rate for ruminants fed at low output levels, which was 0.05 h^−1^ (25).

### *In vivo* experiment

#### Animals, experimental design, and treatments

Six crossbred heifers (*Bos taurus* × *Bos indicus*) with average body weight (BW) of 383 ± 21.6 kg (mean ± SD) were used in this experiment. The heifers were randomly assigned to three treatments (no additive [CTL], 0.25% OEO [OEO1], and 0.5% OEO [OEO2]) in a replicated 3 × 3 (*n* = 6) Latin square design. Each period lasted for 21 days. The acclimation period was between 1 and 14 days. Measurements were taken between day 15 and day 21. The heifers were acclimated to indirect open-circuit respiration chambers for CH_4_ measurements before the beginning of the experiment.

#### Feed intake and apparent total tract digestibility

Heifers were provided unlimited access to water and fed the TMR (Table 1) at 2.8% of their body weight. Heifers were fed once daily at 0800 h. The TMR was formulated to satisfy the energy and protein requirements for maintenance and growth (25). The difference between the amount of feed supplied and that rejected the next day was used to compute daily feed intake. Between day 17 and day 21 of each period, the total amount of feces was collected in metabolic crates to determine the apparent total tract digestibility of nutrients. A sub-sample of 10% of the total feces was taken after homogenization. The fecal samples were dried in a forced-air oven (55°C for 72 h) and ground through a 1-mm screen (Willey mill, Arthur H. Thomas Co., Philadelphia, PA, USA) before chemical analysis. To avoid contamination of the feces with urine, each of the metabolic cages had a metal grid that allows urine and feces to pass through, but the heifers stayed dry and comfortable. Below the first grid, two steel mesh grills with 11.5-mm diameter holes collected feces and permitted the passage of urine into a container.

#### Rumen fermentation parameters

Five hours after feeding on day 18 of each period, 8 mL of ruminal liquor was collected using a syringe connected to a stainless-steel tube through the rumen cannula (20-mm internal diameter), to which 2 mL of 25% of metaphosphoric acid was added. The samples were then stored at −20°C before the determination of VFA content by gas chromatography (7820A GC system Agilent Technologies Inc, Santa Clara, CA, USA). An additional sample (50 mL) of rumen liquor was taken to measure the pH using a portable pH meter (Hannah^®^ Instruments, Woonsocket, RI, USA).

#### Methane production

Methane measurements were carried out in two open-circuit respiration chambers with an internal volume of 9.97 m^3^. Each chamber was equipped with a steel feeder (90 cm length, 70 cm height, and 50 cm width), automatic bowl-type waterer (20 cm diameter), fan (30 cm diameter), air conditioner (12,000-BTU), and dehumidifier. The temperature and relative humidity were maintained at 23 ± 1°C and 55 ± 10%, respectively. A polyvinyl chloride tube and a half-turn valve (75-mm diameter) positioned in the upper-right corner of the front wall regulated the supply of ambient air and air pressure within the chamber (−275 Pa). Ambient air was drawn from the chambers by mass flowmeters (Flowkit; Sable Systems International; Las Vegas, NV, USA), adjusted to the animal's body weight (1 L min/kg BW), and sent to the sample device using a flexible industrial pipe (50-mm diameter). A CH_4_ analyzer that uses infrared light (MA-10 Sable Systems International^®^, Las Vegas, NV, USA) and respiration chambers were calibrated by infusing a known amount of pure CH_4_ (Praxair^®^ Gases Industrial Inc., Monterrey, NL, Mexico; 99.997% purity), with recovery values ranging from 97 to 102%. Every morning before each run, the calibration was verified by zeroing the apparatus with pure N_2_ (Praxair^®^, State of Mexico, Mexico) and then releasing CH_4_ (1000 ppm, Praxair^®^, State of Mexico, Mexico) diluted in N_2_ until stable readings (plateau) were obtained after 5 min. The measurements were carried out for three consecutive days (day 19 to day 21 of each period), and chambers were cleaned in 1 h. Expe Data^®^ software was used to extrapolate the data to a 24 h period (26). According to IPCC (27), GEI/day lost as CH_4_ was computed from its heat of combustion (CH_4_ = 55.65 MJ/kg).

#### Chemical analyses

The total feed provided, refused feed, and fecal samples were oven-dried for 72 h at 60°C, ground through a 1-mm screen (Wiley mill; A. H. Thomas, Philadelphia, PA, USA), and analyzed for DM (method 930.15), ash (method 923.03), and crude protein (CP) using a C/N-analyzer (CN-2000 series 3740, LECO^®^ Corp., St. Joseph, MI, USA; AOAC, method number 992.15), as well as ether extract (EE, AOAC, method number 920.39). An adiabatic bomb calorimeter was used to determine gross energy (model 6400 Parr Instrument Company, Moline, IL, USA). The concentrations of NDF and acid detergent fiber (ADF) (AN 3805 ANKOM, ANKOM Technology, Wayne County, NY, USA) were determined following the method proposed by Van Soest et al. (28). Briefly, NDF and ADF were determined using ~0.5-g samples with fiber analysis bags (F57). Samples were treated with a neutral detergent solution and rinsed with a heat-stable amylase to remove the soluble non-structural carbohydrates from the matrix; the remaining compounds were cell wall compounds, such as cellulose, hemicellulose, and lignin. Thereafter, hemicellulose was removed from the matrix by making it soluble with an acid detergent solution. Cellulose and lignin remaining in the matrix were treated with sulfuric acid to remove cellulose using an ANKOM^200^ fiber analysis system and commercially available solutions (ANKOM Technology, Wayne County, NY, USA).

### Statistical analyses

All data from the *in vitro* studies were analyzed using the GLM procedure in SAS (version 9.4; SAS Inst., Inc., Cary, NC, USA) for a completely randomized design. The model used was as follows:


Y = μ+Ti+e

where μ is the overall mean, Ti is the treatment effect, and *e* is the error term. Least-squares means tests were used to determine the standard error of the difference between means. Tukey tests were used to compare means. Polynomial contrasts were performed to evaluate the linear and quadratic effects of the treatments. Data from the *in vivo* experiment were subjected to analysis of variance for a replicated 3 × 3 Latin square design using the mixed procedure in the SAS^®^ 9.4 Software (29). The statistical model was as follows:


Yijk = μ+Pi +Aj +Tk+Eijk

where *Y* is the dependent variable, μ is the general mean, *P* is the effect of period, *A* is the random effect of animal, *T* is the effect of treatment, and *E* is the random residual error. Results were compared using least-squares means tests, and polynomial contrasts were used to assess the treatment effect.

Kinetics of degradation and potential rumen degradability *in situ* were obtained from the equation proposed by Ørskov and McDonald (24) for each treatment using the non-linear Marquardt procedure of SAS (version 9.4; SAS Inst., Inc., Cary, NC, USA), and the *in situ* degradation kinetic parameters were analyzed using the mixed procedure in SAS (version 9.4; SAS Inst., Inc.), in which treatment was a fixed effect and incubation replicate in the rumen was a random effect. The model used for the analysis was as follows:


Yij = μ+Fi +Rj +eij

where *Y* = the observation of the dependent variables *ij*; μ = the overall mean of *Y*; *Fij*= the effect of treatment (*i* = 3), *R* = the effect of incubation run as replicate (*j* =6 animals), and *eij* = the random error associated with the observation *ij*. Standard error of the difference among means was carried out using least-squares means tests.

## Results

### Volatile composition of OEO

The main volatile compounds identified in OEO are shown in Table 2. The compounds representing more than 99% of the total composition of OEO were D-limonene (78.84%), β-myrcene (6.55%), α-pinene (2.4%), linalool (1.61%), sabinene (1.17%), and β-phellandrene (1.08%).

**Table 2:** Volatile composition of orange essential oil (OEO).

**RI**	**Compound**	**%**
1200	D-Limonene	78.84
1161	β-Myrcene	6.55
1026	α-Pinene	2.40
1547	Linalool	1.61
1124	Sabinene	1.17
1210	β-Phellandrene	1.08
1497	Decanal	0.87
1289	Octanal	0.64
1111	β-Pinene	0.57
1246	γ-Terpinene	0.49
1725	Geranial	0.43
1727	β-Bisabolene	0.36
1697	α-Terpineol	0.35
1147	3-Carene	0.32
1679	Neral	0.23
1598	trans-β-Caryophyllene	0.21
1712	Dodecanal	0.21
1272	p-Cymene	0.20
1755	δ-Cadinene	0.20
1477	β-Citronellal	0.20
1556	1-Octanol	0.15
2237	β-Sinensal	0.15
1710	D-Germacrene	0.14
1738	Carvone	0.13
1167	α-Phellandrene	0.13
1391	Nonanal	0.12
1492	α-Copaene	0.12
1793	Perillaldehyde	0.12
1597	β-Copaene	0.11
1639	(E)-p-Menth-2,8-dienol	0.11
1461	trans-Limonene oxide	0.11
1450	cis-Limonene oxide	0.09
1667	α-Humulene	0.08
1283	α-Terpinolene	0.08
1665	(E)-β-Farnesene	0.07
1754	1-Decanol	0.07
2304	α-Sinensal	0.07
1557	cis-α-Bergamotene	0.06
1727	α-Farnesene	0.06
1475	Octyl acetate	0.06
1845	trans-Carveol	0.06
-	p-Mentha-1(7),8(10)-dien-9-ol	0.05
1666	(Z)-p-Menth-2,8-dienol	0.05
-	Perilla acetate	0.04
1856	cis-Carveol	0.04
-	p-Mentha-1,8-dien-3-one, (+)-	0.03
1794	Nerol	0.03
1591	β-Elemene	0.03
1845	Geraniol	0.03
1987	β-Caryophyllene oxide	0.03
2057	Germacrene D-4-ol	0.03
1660	1-Nonanol	0.02
2135	Hexadecanal	0.02
2080	Elemol	0.02
1069	Camphene	0.02
1957	Cubebol	0.02
1810	2,4-Decadienal	0.02
-	m-Camphorene	0.02
1235	cis-β-Ocimene	0.01
1972	cis-Caryophyllene epoxide	0.01

RI, retention indices reported on the polar column Babushok, V. I., Linstrom, P. J., & Zenkevich, I. G. (62) and NIST Chemistry WebBook (63).

### *In vitro* experiment

#### *In vitro* fermentation patterns

The inclusion of OEO had no effect (*P* > 0.05) on *in vitro* pH, which ranged between 6.7 and 6.8. The proportions of acetate, propionate, isobutyrate, butyrate, isovalerate, and valerate were not altered by OEO supplementation (*P* > 0.05); the acetate:propionate ratio ranged from 1.9 to 2.0 (Table 3) and was also not affected by OEO supplementation.

**Table 3:** Effect of orange essential oil (OEO) on pH and volatile fatty acids *in vitro* (*n* = 6).

	**Treatment**				***P*** **-value**		
**Item**	**CTL**	**OEO1**	**OEO2**	**SEM**	**Type 3**	**linear**	**quad**
pH	6.8 ± 0.05	6.7 ± 0.03	6.8 ± 0.03	0.03	0.3243		
**Volatile fatty acids**
Acetate (%)	44.63 ± 1.4	46.5 ± 1.2	43.8 ± 1.0	0.74	0.3351	0.6465	0.1719
Propionate (%)	23.4 ± 0.19	23.6 ± 0.26	22.2 ± 0.5	0.27	0.0664	0.0638	0.1038
Butyrate (%)	20.0 ± 0.8	18.9 ± 0.4	21.1 ± 0.41	0.43	0.0852	0.2192	0.0534
Isobutyrate (%)	3.6 ± 0.16	2.9 ± 0.09	3.5 ± 0.31	0.15	0.1297	0.6433	0.0512
Isovalerate (%)	5.5 ± 0.36	4.7 ± 0.07	5.3 ± 0.34	0.19	0.2264	0.5865	0.1082
Valerate (%)	2.9 ± 0.20	3.3 ± 0.85	4.1 ± 0.79	0.38	0.4794	0.2702	0.7952
Acetate:propionate ratio	1.9 ± 0.07	2.0 ± 0.07	2.0 ± 0.03	0.03	0.7489	0.5321	0.6961

CTL, control; OEO1, 0.25% OEO in the incubated dry matter (IDM); OEO2, 0.50% IDM; Means (± SD from n = 6).

#### Gas and methane production

No differences in *G*_max_ (mL/g IDM) and GP24 (mL/g IDM) between the OEO treatments and the control were observed (*P* > 0.05; Table 4). However, *A* (h) and *K* (% h^−1^) were higher in the OEO1 and OEO2 treatments compared with the control (*P* < 0.05). The production of CH_4_ decreased by 41.7% (mL at 24 h), 41.6% (mL/g IDM), and 45.9% (mL/g degraded DM), respectively, when OEO was supplemented at 0.25% DM (*P* < 0.05). There was a quadratic relationship between the dose of OEO and CH_4_ production (ml/g IDM and ml/g of degraded DM; *P* < 0.05).

**Table 4:** Maximum *in vitro* gas production, lag phase, constant gas production rate, and *in vitro* CH_4_ production in response to supplementation with orange essential oil (OEO; *n* = 6).

	**Treatment**				***P*** **-value**		
**Item**	**CTL**	**OEO1**	**OEO2**	**SEM**	**Type 3**	**linear**	**quad**
Gmax (mL/g IDM)	115.2 ± 1.5^a^	81.0 ± 2.8^a^	144.5 ± 21^a^	12.9	0.0825	0.1979	0.0501
A (h)	1.83 ± 1^−3*c*^	2.45 ± 3^−2*a*^	2.3 ± 5^−3*b*^	0.12	<0.001	<0.001	<0.001
K (% h^−1^)	0.06 ± 1^−3*c*^	0.24 ± 1^−2*a*^	0.15 ± 1^−2*b*^	0.03	0.0016	0.0035	0.0012
GP24 (mL/g IDM)	67.2 ± 0.9^a^	78.1 ± 3.3^a^	122.2 ± 17^a^	11.5	0.0550	0.0284	0.2604
CH_4_ (mL at 24 h)	6.27 ± 0.1^a^	3.65 ± 0.3^b^	7.03 ± 0.5^a^	0.54	<0.001	0.2660	<0.001
CH_4_ (mL/g IDM)	12.5 ± 0.3^a^	7.3 ± 0.6^b^	14.1 ± 0.9^a^	1.08	<0.001	0.2660	<0.001
CH_4_ (mL/g DDM)	23.3 ± 0.8^a^	12.6 ± 2.4^b^	26.2 ± 2.4^a^	2.26	0.0136	0.3104	0.0056
DDM (g/g IDM)	0.54 ± 3^−2^	0.59 ± 3^−2^	0.54 ± 3^−2^	0.02	0.6631	0.9732	0.3884
CO_2_ (mL/g IDM)	54.8 ± 1.5	44.2 ± 1.6	49.7 ± 4.2	2.05	0.0880	0.2415	0.0527

CTL, control; OEO1, 0.25% OEO in the incubated dry matter (IDM); OEO2, 0.50% IDM; Gmax, maximum gas production; A, the lag period before gas production begins (lag phase); K, constant gas production rate; GP24, gas production at 24 h; DDM, degraded dry matter. Means (± SD from n = 6); ^a−c^Means within a row with different superscripts are significantly different (P < 0.05) for type 3 tests of fixed effects; SEM, standard error of the mean.

### *In situ* experiment

There were no changes in the parameters assessed *in situ* (Table 5), including potential and effective degradability in response to OEO supplementation (*P* > 0.05).

**Table 5:** Effect of orange essential oil (OEO) on *in situ* rumen degradation kinetics (*n* = 6).

**Item**	**Treatment**				
	**CTL**	**OEO1**	**OEO2**	**SEM**	***P*-value**
a (%)	31.62 ± 1.38	30.19 ± 0.50	27.57 ± 2.21	1.53	0.3123
b (%)	38.85 ± 0.29	42.24 ± 4.44	43.35 ± 1.27	2.67	0.5145
c (h^−1^)	0.028 ± 6^−3^	0.024 ± 4^−3^	0.027 ± 2^−3^	0.4^−3^	0.5200
PD (%)	70.47 ± 1.09	72.43 ± 3.93	70.92 ± 3.47	3.09	0.6912
ED (%)	45.15 ± 0.67	44.93 ± 1.03	41.50 ± 1.06	0.94	0.1734

CTL, control; OEO1, 0.25% OEO in the incubated dry matter (IDM); OEO2, 0.50% IDM; a, soluble fraction; b, insoluble but potentially degradable fraction; c, fractional disappearance rate constant at which b is degraded; PD, potential degradability; ED, effective degradability; Means (± SD from n = 6); ^a−c^Means within a row with different superscripts are significantly different (P < 0.05) for type 3 tests of fixed effects; SEM, standard error of the mean.

### *In vivo* experiment

#### Feed intake and apparent total tract digestibility

Supplementation of OEO had no effect on DM, OM, CP, NDF, and ADF intake (*P* > 0.05; Table 6); however, there was a negative quadratic relationship between GE intake and the level of OEO supplementation (*P* < 0.05; Table 6). The apparent total tract digestibility of DM decreased quadratically (*P* < 0.05; Table 7) when 0.5% OEO was added. The apparent total tract digestibility of OM, CP, NDF, and ADF and digestible energy were not affected (*P* > 0.05) by OEO supplementation.

**Table 6:** Effect of orange essential oil (OEO) supplementation on the feed intake of heifers (*n* = 6).

	**Treatment**				***P*** **-value**		
**Item**	**CTL**	**OEO1**	**OEO2**	**SEM**	**Type 3**	**linear**	**quad**
DMI (kg/d)	9.38	9.76	9.11	0.75	0.1471	0.2285	0.1107
OM (kg/d)	8.73	8.98	8.30	0.68	0.0932	0.3673	0.0460
CP (kg/d)	0.74	0.75	0.64	0.07	0.4416	0.8930	0.2012
NDF (kg/d)	6.87	7.21	7.29	0.57	0.5490	0.4105	0.4835
ADF (kg/d)	4.87	5.02	4.65	0.37	0.4391	0.6051	0.2500
GE (MJ/d)	143.6^ab^	150.7 ^a^	137.3 ^b^	11.42	0.0479	0.1495	0.0336

CTL, control; OEO1, 0.25% dry matter intake (DMI); OEO2, 0.50% DMI; OM, organic matter; CP, crude protein; NDF, neutral detergent fiber; ADF, acid detergent fiber; GE, gross energy. ^a, b^Means within a row with different superscripts are significantly different (P < 0.05) for type 3 tests of fixed effects; SEM, standard error of the mean.

**Table 7:** Effect of orange essential oil (OEO) on the apparent total tract digestibility of heifers (*n* = 6).

	**Treatment**				***P*** **-value**		
**Item**	**CTL**	**OEO1**	**OEO2**	**SEM**	**Type 3**	**linear**	**quad**
DM (g/kg)	653.33 ^a^	658.04 ^a^	613.32 ^b^	10.08	0.0117	0.7088	0.0038
OM (g/kg DMI)	643.04	641.46	612.74	10.10	0.0703	0.9023	0.0500
CP (g/kg DMI)	61.77	57.78	52.21	7.03	0.6309	0.6928	0.3948
NDF (g/kg DMI)	469.30	476.14	531.69	22.34	0.1002	0.8095	0.0500
ADF (g/kg DMI)	3.45	3.36	3.14	20.79	0.4183	0.6942	0.2187
Digestible energy (MJ/kg DMI)	10.47	10.60	9.76	0.25	0.0933	0.7229	0.0502

CTL, control; OEO1, 0.25% dry matter intake (DMI); OEO2, 0.50% DMI; OM, organic matter; CP, crude protein; NDF, neutral detergent fiber; ADF, acid detergent fiber. ^a, b^Means within a row with different superscripts are significantly different (P < 0.05) for type 3 tests of fixed effects; SEM, standard error of the mean.

#### Rumen fermentation parameters

Rumen pH, VFA, and the acetate:propionate ratio are shown in Table 8. Rumen pH was similar across treatments (*P* > 0.05) and ranged from 6.57 to 6.68. There was a quadratic response in acetate proportion when OEO was added (*P* < 0.05). The proportion of valerate decreased quadratically in response to OEO addition (*P* < 0.05). Feeding OEO1 resulted in an increase of valerate by 21% relative to the control, whereas valerate decreased by 14% in response to OEO2 compared to the control (*P* < 0.05). Supplementation of OEO had no effect on the C3:C2 ratio (*P* > 0.05).

**Table 8:** Effect of orange essential oil (OEO) on rumen pH and volatile fatty acid (VFA) production in heifers (*n* = 6).

	**Treatment**				***P*** **-value**		
**Item**	**CTL**	**OEO1**	**OEO2**	**SEM**	**Type 3**	**linear**	**quad**
pH	6.68	6.67	6.57	0.09	0.6427	0.9015	0.3659
**Volatile fatty acids**
Acetate (%)	48.96	49.18	50.38	0.41	0.0779	0.7120	0.0501
Propionate (%)	24.22	23.76	23.24	0.39	0.2662	0.4299	0.1564
Butyrate (%)	18.72	19.70	19.07	0.65	0.4228	0.2087	0.8264
Isobutyrate (%)	2.06	2.16	1.86	0.16	0.4283	0.6734	0.2288
Isovalerate (%)	3.39	3.40	3.19	0.21	0.7311	0.9645	0.4436
Valerate (%)	2.64 ^ab^	3.21 ^a^	2.27 ^b^	0.18	0.0155	0.0501	0.0156
Acetate:propionate ratio	2.03	2.08	2.18	0.04	0.0797	0.3959	0.0503

CTL, control; OEO1, 0.25% dry matter intake (DMI); OEO2, 0.50% DMI. ^a, b^Means within a row with different superscripts are significantly different (P < 0.05) for type 3 tests of fixed effects; SEM, standard error of the means.

#### Methane production

When 0.5% OEO was fed, CH_4_ (g/day) was reduced by 12% as compared to control (*P* < 0.05; Table 9). The CH_4_ yield (g/kg DMI) decreased linearly with the level of OEO supplementation. No changes were observed in emissions per kilogram of fermented OM in the rumen (*P* > 0.05). There was a linear decrease in CH_4_ yield (% GE/day) in response to OEO addition. There was a quadratic decrease in energy loss in form of CH_4_ (MJ of GEI/day) in response to OEO2 supplementation (*P* < 0.05).

**Table 9:** Effect of orange essential oil (OEO) on the enteric CH_4_ production of heifers (*n* = 6).

	**Treatment**				***P*** **-value**		
**Item**	**CTL**	**OEO1**	**OEO2**	**SEM**	**Type 3**	**linear**	**quad**
CH_4_ (g/d)	139.58 ^a^	137.10 ^a^	122.72 ^b^	7.83	0.0152	0.6156	0.0052
CH_4_ (g/kg DMI)	16.33	14.30	13.43	0.75	0.0647	0.0923	0.0743
CH_4_ (g/kg fermented OM)	36.09	34.23	33.10	1.24	0.1206	0.1837	0.1039
CH_4_ **(**MJ of GEI/d)	7.77 ^a^	7.63 ^a^	6.83 ^b^	0.44	0.0152	0.6156	0.0052
Ym (% GEI/d)	5.98	5.15	4.96	0.27	0.0791	0.0792	0.1192

CH_4_ g/day; CH_4_ g/kg DMI, CH_4_ g/kg dry matter intake; OM, organic matter; GEI, gross energy intake; Ym, CH_4_ MJ/day, expressed as percentage of gross energy intake (GEI). ^a, b^Means within a row with different superscripts are significantly different (P < 0.05) for type 3 tests of fixed effects; SEM: standard error of the mean.

## Discussion

### Volatile composition of OEO

The composition of volatile compounds in OEO was consistent with that reported in previous studies (30–33); D-limonene, β-myrcene, and α-pinene were the most abundant volatile compounds. Variation in the concentrations of volatile compounds is associated with variation in species, geographical origin, climatic conditions, extraction technique, and fruit maturity (34).

### *In vitro* experiment

#### *In vitro* fermentation patterns

Changes in rumen pH in our study were consistent with those observed by Castillejos et al. (35), who used the most abundant monocyclic monoterpene in OEO (limonene) in doses of 5–500 mg/L rumen liquor in a basal diet with 60:40 forage:concentrate ratio. However, our findings differ from those of Kamalak et al. (16), who found that increases in OEO supplementation (200, 400, 800, and 1,200 mg/L) increase rumen pH. In our experiment, VFA (% of total) was not significantly affected by OEO supplementation, suggesting that the doses evaluated did not affect rumen bacteria (OEO1: 2,500 mg/L and OEO2: 5,000 mg/L). Kamalak et al. (16), who used rumen fluid from sheep, reported that high doses of OEO (800 and 1,200 mg/L) reduce the production of VFA (mmol/L) and alter the molar proportion of VFA; specifically, high OEO doses increase the proportion of acetate and the acetate:propionate ratio. Castillejos et al. (36) found that EO supplementation of a diet containing 10% barley straw and 90% concentrate with thyme oil (*Thymus vulgaris*), savory oil (*Satureja montana*), and Lavandin oil (*Lavandula hybrida*) lowered the pH. Furthermore, they found that clover leaf oil (*Eugenia caryophyllus*) and oregano oil (*Origanum vulgare*) increased pH when supplied at 500 mg.

#### *In vitro* gas and methane production

Essential oils, which are typically obtained by steam distillation, are known to have antibacterial properties due to their ability to modify the permeability of cells (37, 38). A previous study (16) showed that OEO supplementation at doses ranging from 100 to 1,200 mg/L reduces CH_4_ production (mmol/L), which is consistent with the results of our study. Methane production was reduced by 41.7% (mL), 41.6% (mL/g incubated DM), and 45.9% (mL/g degraded DM) in response to OEO1. García-Rodríguez et al. (18) evaluated the effect of substituting extruded corn for dry citrus pulp (20%) and found that reductions in CH_4_ were associated with the antimicrobial effects of bioactive compounds such as terpenoids, limonene, and citral. However, this was not observed for the OEO2 treatment, as there were no differences in CH_4_ between OEO2 and the control. The quadratic response in CH_4_ production might stem from the fact that a higher concentration of OEO might have induced toxicity to the microbiota. This finding confirms that the effects of EO on ruminal microbiota are dose-dependent. An alternative explanation for the differences between the results of our study and the results of previous studies might be associated with differences in the concentrations of bioactive compounds, the substrates used (*i.e.*, soybean meal vs. forage: concentrate:forage ratio 70:30), and the techniques used for quantification.

### *In situ* experiment

Few studies have examined the effects of OEO on the *in situ* digestibility of DM. Previous studies of other EOs such as lemongrass oil and a mixture of garlic and ginger oil have shown that supplementation of these EOs (200 mg/kg) enhances the *in situ* DM digestibility of grass hay a TMR (39). Studies using different EOs reported inconsistent findings regarding the degradation of protein-rich substrates using dacron bags in the rumen. This might be due to differences in substrate composition (40). However, in this study, the level of OEO supplementation did not appear to affect *in situ* rumen degradation kinetics.

### *In vivo* experiment

#### Feed intake and apparent digestibility

Increases in the level of OEO supplementation did not affect DMI as has been shown in a previous study that applied 200–1,200 ppm of OEO in a swamp grass:concentrate ratio 60:40 (41). A different study found that supplementation of 4.5 g of citrus extract per day did not improve the DMI of the dairy cows (42). Other EOs have been reported to increase feed intake (carvacrol, eugenol, thymol, coriander seed oil, geranyl acetate, and geraniol), but some studies have demonstrated that blends of thymol, eugenol, carvacrol, garlic, citral, and cinnamaldehyde can induce reductions in feed intake and digestibility (43, 44). This suggests that the effects of different EOs or their constituents on feed intake are variable and will depend on the type and dosage of EO (37). Other studies have suggested that the palatability of feed can be impacted by the addition of EO; however, other factors that also affect DMI—such as animal growth stage, body weight, or the specific properties of diets such as the fiber content and particle size—also require consideration (41, 45, 46). One possible explanation for the reduced rumen degradability associated with the OEO2 treatment might be the broad and non-specific antimicrobial capacity of OEO on rumen microorganisms. The use of 0.5% OEO reduced rumen microbial activity, which affected the fermentability, degradability, and CH_4_ emissions (47). This is consistent with findings of *in vitro* studies showing that 200 and 300 ppm of orange peel oil inhibits CH_4_ generation by more than 50% due to the low digestibility of DM and NDF, which was possibly associated with a microbial imbalance in the rumen (48). The discrepancies among studies might be caused by different types of EOs that were used in the respective studies, dosages tested, and differences in diet and host interactions (49).

### Rumen fermentation parameters

In ruminants fed low-quality tropical grasses as a basal ration, the rate and extent of fermentation of OM in the rumen are usually reduced, which helps maintain pH within the physiological range. Some have suggested that EOs have stronger effects in animals fed high concentrations of grains as in intensive systems; given that bacteria that grow under low pH are favored under such conditions, limonene has been reported to reduce *Fusobacterium necrophorum* populations as well as the prevalence of liver abscesses (50, 51). The rumen pH was not affected by OEO supplementation (41), and it remained within the optimal range for fermentation (6.6 ± 0.5) (52). The above findings are also consistent with the results of other studies that have used a mixture (750 mg/day) of EOs (thymol, eugenol, vanillin, guaiacol, and limonene) (53). Afzalani et al. (41) found that the use of 200 and 400 ppm OEO in cattle increased the concentration of propionic acid in the rumen and affected the C3:C2 ratio. No changes in the rumen microbial catabolism of branched-chain amino acids were observed in response to OEO supplementation; the concentration of valerate increased with the level of OEO1 supplementation, which indicates that valerate acted as a sink for H_2_ and contributed to reductions in CH_4_ (54). Wu et al. (2018) suggested that EO can affect rumen fermentation by inhibiting microbial growth.

### Methane production

Previous authors who supplemented EO and detected reductions in enteric CH_4_ suggested the following reasons for the observed reduction: (1) a reduction in hydrogen production (alternative sinks), (2) the direct inhibition of archaea, and (3) a disruption of the symbiosis between protozoa and archaea (55). Few studies of the effects of EO and OEO from *Citrus sinensis* have been conducted (2). Wu et al. (17) reported that citrus EO (limonene) had anti-methanogenic effects in Hu sheep, but this effect was not consistent among periods, suggesting that microbial adaptation can occur following short-term exposure to EO. Phytogenic feed additives, such as for example Agolin Ruminant (Agolin, Bière, Switzerland) or Mootral (Mootral SA, Rolle Switzerland), are often mixtures of different plant extracts containing EOs. The fact that commercial phytogenic additives are typically complex mixtures of different compounds, makes it challenging to identify the bioactive compounds responsible for beneficial effects like CH_4_ reduction. A large amount of bioactive compounds in those additives makes it challenging to identify their mode of action and predict the effectiveness of those mixtures when added to different basal diets. In this study, a trend for a quadratic response in CH_4_ (g/day) for an increase in OEO supplementation was detected. The use of high doses of OEO with antimicrobial activity likely decreased the microbial activity and fermentability of the diet. It has been described that the interactions among different components in EOs may affect their antimicrobial activity (56). The availability of raw materials is also important, although this is not a problem for OEO given that it can be obtained from one of the most common subtropical crops in the world (13, 57). The quality of meat is not affected by EO (58, 59). The inclusion of OEO in the diet of sheep has been shown to improve milk yield, fat milk yield, and feed efficiency (11). In our study, discrepancies between *in vitro* and *in vivo* results might be caused by the absence of VFA absorption and passage in *in vitro* batch systems compared to *in vivo*, as well as differences in the composition of the microbial population (60, 61). Our findings demonstrate that OEO decreased CH_4_ emissions, which also might lead to improved feed efficiency (2).

## Conclusion

Orange essential oil, containing 78.84% D-limonene, supplemented at 0.25% did not have adverse effects on DMI, apparent total tract digestibility, rumen fermentation parameters, or enteric CH_4_ production. Supplementation of 0.5% OEO reduced CH_4_ emissions by 12%; however, 0.5% OEO had a negative effect on the apparent total tract digestibility of DM.

## Data availability statement

The raw data supporting the conclusions of this article will be made available by the authors, without undue reservation.

## Ethics statement

The animal study was reviewed and approved by Bioethics Committee at the University of Yucatan (approval no. CB-CCBA-D-2019-01).

## Author contributions

RJ-O, MM-F, EA-R, and ME-E: investigation, writing-original draft, review, editing, conceptualization, and data elaboration. JK-V and JA: supervision, funding acquisition, and review and editing. EH-T, MG-C, EA-R, and GP-C: *in vitro* experiments, writing-review and editing, and data elaboration. CA-P: review, editing, validation, and data elaboration. All authors contributed to the editing of the paper and the intellectual content, and have approved it for publication.

## Funding

This research was funded by the one CGIAR initiative on Livestock, Climate, and System Resilience (LCSR). We thank all donors that globally Support our work through their contributions to the CGIAR system.

## Conflict of interest

The authors declare that the research was conducted in the absence of any commercial or financial relationships that could be construed as a potential conflict of interest.

## Publisher's note

All claims expressed in this article are solely those of the authors and do not necessarily represent those of their affiliated organizations, or those of the publisher, the editors and the reviewers. Any product that may be evaluated in this article, or claim that may be made by its manufacturer, is not guaranteed or endorsed by the publisher.
